# Knowledge and Beliefs of Breast Self-Examination and Breast Cancer among Market Women in Ibadan, South West, Nigeria

**DOI:** 10.1371/journal.pone.0140904

**Published:** 2015-11-25

**Authors:** Kelechi Elizabeth Oladimeji, Joyce M. Tsoka-Gwegweni, Franklin C. Igbodekwe, Mary Twomey, Christopher Akolo, Hadiza Sabuwa Balarabe, Olayinka Atilola, Oluwole Jegede, Olanrewaju Oladimeji

**Affiliations:** 1 Discipline of Public Health Medicine, College of Health Science, Howard College Campus, University of KwaZulu-Natal, Durban, South Africa; 2 Center for Community Healthcare, Research and Development, Abuja, Nigeria; 3 Health and Social Care Department, Open University, Milton Keynes MK7 6BJ, London, United Kingdom; 4 Population Services International, 1120 19^th^ Street, N.W, Suite 600, Washington, District of Columbia, 20036, United States of America; 5 Department of Public Health and Human Services, Federal Capital Territory, Abuja, Nigeria; 6 Department of Psychiatry, Lagos State University, Lagos, Nigeria; 7 Milken Institute of Public Health, George Washington University, Washington, District of Columbia, 20052, United State of America; University of South Alabama Mitchell Cancer Institute, UNITED STATES

## Abstract

**Background:**

In most resource constrained settings like Nigeria, breast self-examination self-breast examination (BSE) is culturally acceptable, religious friendly and attracts no cost. Women's knowledge and beliefs about breast cancer and its management may contribute significantly to medical help-seeking behaviours. This study aimed to assess knowledge and beliefs of BSE among market women.

**Methods:**

A descriptive cross-sectional study was conducted among 603 market women in Ibadan, Nigeria. Data was collected using semi-structured interviews and analyzed using descriptive and analytic statistical methods.

**Results:**

The mean age of the respondents was 34.6±9.3 years with 40% of the women aged between 30-39years. The proportion of married women was 339 (68.5%) with 425 (70.8%) respondents reporting that they do not know how to perform BSE. However, 372 (61.7%) women strongly agreed that BSE is a method of screening for breast cancer. Highest proportion 219 (36.3%) reported that the best time for a woman to perform BSE was ‘anytime’. Most of the respondents believed breast cancer is a dangerous disease that kills fast and requires a lot of money for treatment.

**Conclusion:**

More efforts are needed in creating awareness and advocacy campaigns in the grassroots in order to detect early breast cancer and enhance prevention strategies that would reduce the burden of breast cancer in Nigeria.

## Introduction

Breast cancer is a global health concern and a leading cause of morbidity and mortality among all the cancers that affect women [1]. In 2008, it was estimated that the prevalence of breast cancer in women aged 15 years and over in Sub-Saharan Africa is 23.5 per 100,000 women [2]. Breast cancer has been identified as a major public health problem in both developed and developing nations because of its high incidence-prevalence, the over-burdened health system and direct medical expenditure [3]. Global statistics shows that the annual incidence of breast cancer is increasing and this is occurring more rapidly in countries with a low incidence rate of breast cancer [4–5]. Findings from Elima Jedy-Agba et al. in 2012 [6] documented that the incidence of breast cancer in Nigeria has risen significantly with incidence in 2009–2010 at 54.3 per 100 000, thereby representing a hundred percent increase in the last decade. Some cases have been reported among women aged below 30 years in Nigeria [7]. This is supported by the literature showing a rise in breast cancer incidence rates in Sub-Saharan Africa [8].

The high incidence of breast cancer necessitates the need for early detection because this would increase the treatment options available to affected women and thereby improve survival rates [9]. Some studies have shown that in most of the developing nations and resource constraint settings, breast cancer is diagnosed in advanced stages of the disease when compared with developed nations and thus has a poor outcome and high fatality rate [1, 10–17].

Screening for early detection and diagnosis of diseases and health conditions is an important public health principle [18]. Breast self-examination (BSE) is a check-up that a woman does by herself at home to look for changes or problems affecting the breast tissue. BSE is still recommended as a general approach to increasing breast health awareness and thus potentially allow for early detection of any anomalies because it is free, painless and easy to practice [19]. The American Cancer Society [20] also recommends that women, starting from the age of 20 years should be educated on the pros and cons of performing a monthly BSE. For women to present early to hospital they need to be "breast aware"; they must be able to recognize symptoms of breast cancer [21].

There are reports suggesting that factors related to women's knowledge and beliefs about breast cancer and its management may contribute significantly to medical help-seeking behaviours [21–22]. Recent studies in Senegal, Angola and Nigeria [23–29] revealed a low level of awareness and knowledge on breast cancer risk factors and its early warning signs. Lack of understanding of the risk factors associated with breast cancer discourages people from seeking early intervention or even to admit that symptoms they may be experiencing are related to breast cancer. As such there is need for a study to assess knowledge and beliefs about breast examination BSE and risk factors among women in our communities. This study therefore aimed to assess the knowledge and beliefs of breast self-examination and breast cancer among market women in densely populated markets in Ibadan, Oyo State, Nigeria.

## Methods

### Ethics

Ethical approval was given by the Oyo state, Ministry of Health Ethics Committee in August, 2012. Participant information was anonymized and de-identified prior to analysis. Informed consent was obtained from the participants aged 15 and above. The named ethics committees approved the consent procedure in addition to the study protocol.

A cross-sectional study was conducted between July to October 2012, in order to assess knowledge and beliefs on BSE among women selling in a few major markets in Ibadan, Oyo State, Nigeria. Major markets were purposefully selected and subsequently consented participants were interviewed from each of the market. Ibadan is the largest indigenous city south of the Sahara and is the capital of Oyo state, Nigeria. It has a population of about 2.6 million people [30]. The study population comprised of 603 women selling at Oja-Oba, Agbeni, Bode, Oje and other markets in Ibadan. These markets are the major markets in Ibadan. These women constitute eighty-percent of all traders selling in the selected markets. They sold mainly food items such as meat, pepper, vegetables, provisions, raw rice and other food stuffs.

### Sample size calculation

1. Estimate of the expected proportion (*p*) of knowledge of breast self-examination among market women = 0.5
2. Desired level of absolute precision (*d*) = 0.05
3. Estimated design effect (*DEFF*) = 1.5

$$n=\frac{1.96^{2}\;p(1-p)(DEFF)}{d^{2}}$$

$$n=\frac{1.96^{2}\times .5\times .5(1.5)}{.05^{2}}=576.24$$

4. Assuming 4% will declining to participate in the study

Minimum sample size = 576.24 + 23.05 = 599 participants

Data was collected using interviewer administered semi structured questionnaires on socio-demographic characteristics, knowledge, attitude and belief of participants after obtaining written informed consent. Data was entered and analysed using statistical package for social sciences (SPSS) version 20. Descriptive and Chi-square statistics was employed in analysing the data.

## Results

### Socio-demographic profile of participants

A total of 603 market women were recruited. Table 1 shows the socio demographic profile of the respondents. There was a fair distribution of the women recruited at the various markets.The mean age of the respondents was 34.6±9.3 years. The highest proportion was aged between 30–39 years. The majority of the participants were married 497/603 (82.4%).

**Table 1:** Socio-demographic profile of study participants (n = 603).

Variable	Frequency (n)	Percent (%)
**Age group (years)**		
**15–19**	10	1.7
**20–29**	182	30.2
**30–39**	232	38.5
**40–49**	137	22.7
**50–59**	24	4.0
**60+**	14	2.3
**Unknown**	4	0.7
**Marital status**		
**Single**	99	16.4
**Married**	497	82.4
**Widower/Divorcee**	5	0.8
**Unknown**	2	0.3
**Educational level**		
**None**	54	9.0
**Primary**	131	21.8
**Secondary**	337	56.0
**Post-secondary**	79	13.1
**Unknown**	2	0.1

### Knowledge about how to perform BSE

More than three-quarters of the participants responded to knowledge on how to perform BSE.The majority of participants 425 (70.8%) reported that they did not know how to perform BSE, while only 29.2% reported that they do.

Knowledge about how to perform BSE was slightly higher in participants who came from Oja-oba- market (37.6%) followed by those from Agbeni, Bode and other markets with level of knowledge all above 25% except in participants from Oje market. Very few participants were recruited in the ages below 20, and 50 years or above. Therefore the latter will not be considered further in this discussion, only ages 20–49 years will be reported. The percentage of participants who reported that they knew how to perform BSE increased with age in participants up to 49 years; with age group 40–49 years (40.4%) being the highest, followed by 30–39 years (32.0%) then 20–29 years. Married participants had a higher knowledge of how to perform BSE than single participants. Knowledge of BSE was correlated with educational level (Table 2), and there were 8.0% of the women with no formal education who reported they did not know while about close to 7% of the study women who had post-secondary education reported to have knowledge. There was a statistically significant relationship between educational level and knowledge on how to perform BSE (p<0.0001),(Table 2).

**Table 2:** Knowledge about how to perform BSE by socio-demographics of study responders. SBE: self-breast examination

Variable	No (Frequency/%)	Yes (Frequency/%)
**Study site/group (N = 600)**		
**Oja-oba market**	63 (62.4)	38 (37.6)
**Agbeni market**	70 (70.7)	29 (29.3)
**Oje market**	78 (78.0)	22 (22.0)
**Bode market**	71 (71.0)	29 (29.0)
**Other markets**	143 (71.5)	57 (28.5)
**Total**	425 (70.8)	175 (29.2)
**Age group in years (N = 596)**		
**15–19**	9 (90.0)	1 (10.0)
**20–29**	139 (76.8)	42 (23.2)
**30–39**	157 (68.0)	74 (32.0)
**40–49**	81 (59.6)	55 (40.4)
**50–59**	24 (100.0)	0(0)
**60+**	11 (78.6)	3 (21.4)
**Total**	421 (70.6)	175 (29.4)
**Marital status (N = 598)**		
**Single**	81 (82.7)	17 (17.3)
**Married**	339 (68.5)	156 (31.5)
**Widower/Divorcee**	3 (60.0)	2 (40.0)
**Total**	423 (70.7)	175 (29.3)
**Educational level (N = 599)**		
**None**	48 (88.9)	6 (11.1)
**Primary**	95 (72.5)	36 (27.5)
**Secondary**	240(71.6)	95 (28.4)
**Post-secondary**	40 (51.3)	38 (48.7)
**Total**	424 (70.8)	175 (29.2)

### Knowledge about when to perform BSE

In total 271 participants responded to the question of when is the right time to perform BSE. Only 8.1% of these knew correctly that ‘mid-cycle’ was the right time to perform BSE.The highest proportion 219 (80.8%), reported incorrectly that the right time for a woman to perform BSE was ‘anytime’. Although below 10%, a large number of women who knew when to perform BSE came from Oja-oba market (8.3%) compared to the other three markets. However, these differences were not statistically significant. Knowledge about when to perform BSE decreased with increasing age showing a slightly higher level of knowledge in the 20–29 year olds than in the 30–39 and 40–49 year age groups(p<0.00). Compared to married, a double percentage of single women knew when to perform BSE. The level of knowledge about when to perform BSE was higher among post-secondary education (15.1%) while among other groups it was less than 10%. These differences were not statistically significant (Table 3).

**Table 3:** Knowledge about when to perform BSE by socio-demographics of study responders. SBE: self-breast examination

Variable	Anytime (Freq/%)	Before menstruation (Freq/%)	Mid cycle (Freq/%)	During menstruation (Freq/%)
Study site/group (N = 271)				
Oja-oba market	41 (85.4)	1 (2.1)	4 (8.3)	2 (4.2)
Agbeni market	31 (79.5)	5 (12.8)	3 (7.7)	0 (0)
Oje market	24 (80.0)	3 (10.0)	1 (3.3)	2 (6.7)
Bode market	24 (80.0)	2 (6.7)	1 (3.3)	3 (10.0)
Other markets	99 (79.8)	10 (8.1)	13 (10.5)	2 (1.6)
Total	219 (80.8)	21 (7.7)	22 (8.1)	9 (3.3)
Age group in years (N-270)				
15–19	0 (0)	0 (0)	1 (100.0)	0 (0)
20–29	54 (72.0)	7 (9.3)	13 (17.3)	1(1.3)
30–39	78 (79.6)	9 (9.2)	7 (7.1)	4 (4.1)
40–49	58 (87.9)	3 (4.5)	1 (1.5)	4 (6.1)
50–59	17 (100.0)	0 (0)	0 (0)	0 (0)
60+	11 (84.6)	2 (15.4)	0 (0)	0 (0)
Total	218 (80.7)	21 (7.8)	22 (8.1)	9 (3.3)
Marital status (N = 271)				
Single	26 (76.5)	3 (8.8)	5 (14.7)	0 (0)
Married	191 (81.3)	18 (7.7)	17 (7.2)	9 (3.8)
Widower/Divorcee	2 (100.0)	0 (0)	0 (0)	0 (0)
Total	219 (80.8)	21 (7.7)	22 (8.1)	9 (3.8)
Educational level (N = 271)				
None	32 (91.4)	2 (5.7)	1 (2.9)	0 (0)
Primary	41 (85.4)	2 (4.2)	3 (6.3)	2 (4.2)
Secondary	108 (80.0)	12 (4.4)	10 (7.4)	5 (3.7)
Post-secondary	38 (71.7)	5 (1.8)	8 (15.1)	2 (3.8)
Total	219 (80.8)	21 (7.7)	22 (8.1)	9 (3.3)

**Fig 1:**
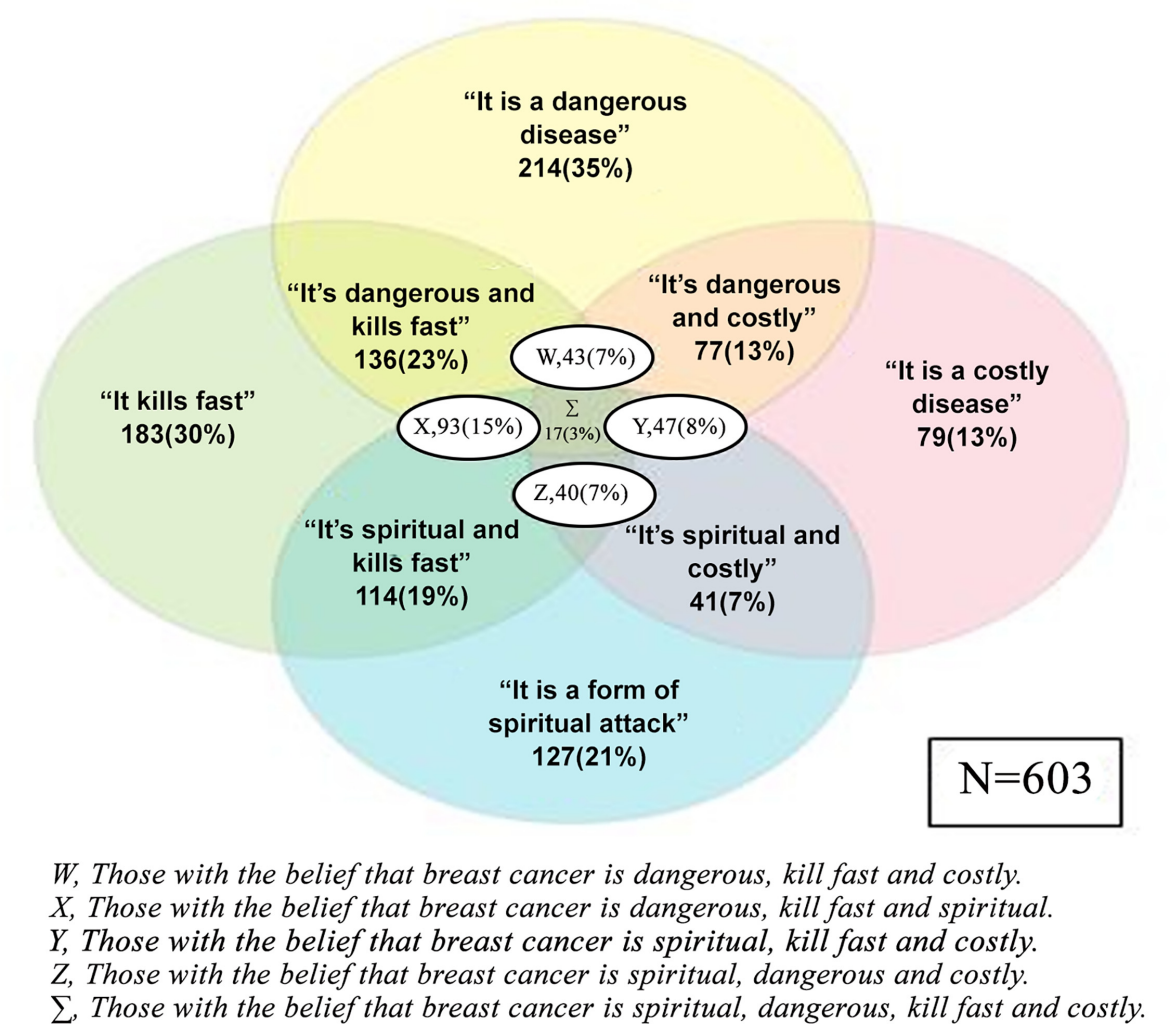
Venn diagram showing beliefs of market women about breast cancer.

**Fig 2:**
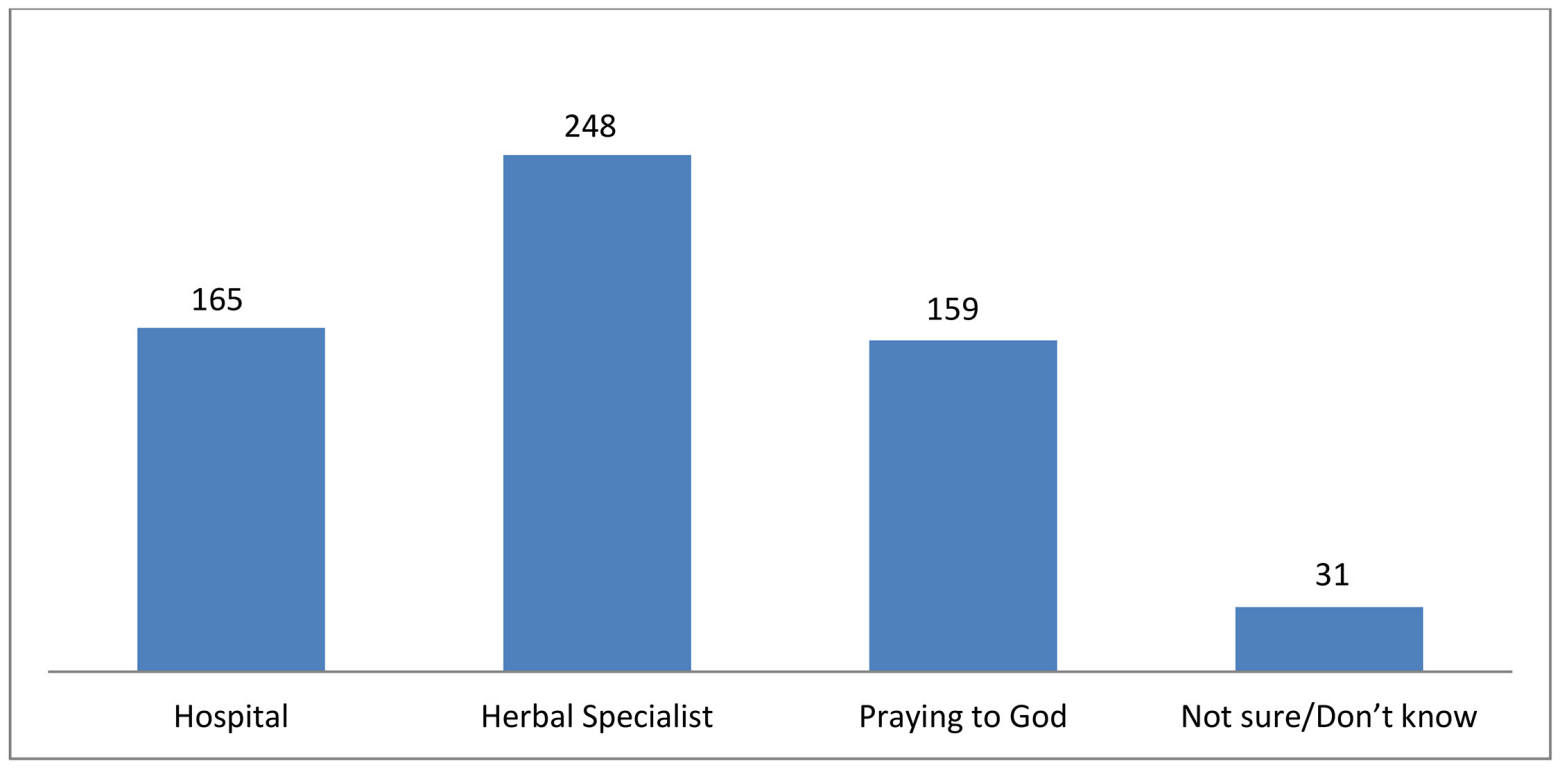
Graph showing the likely referring points of care by the market women.

About two-third (61.7%) of the study population strongly agreed that BSE is a screening method for breast cancer. About 28.5% agreed that fear of detecting breast cancer would make them not practice BSE, while more than 50% strongly disagreed with this statement. The majority of the women strongly disagreed that SBE should be done ‘only if you feel abnormal around your breast’. There was similar responses of participants who strongly agreed or disagreed about postures for SBE (Table 4).When asked about their beliefs on breast cancer, there were varying responses (Fig 1). Many of the respondents had a fair knowledge on the effects of the burden of breast cancer and that the hospital was the place they would refer someone for diagnosis and treatment options (Fig 2).

**Table 4:** Distribution of the study respondents according to their knowledge, attitude and beliefs about breast self-examination (n = 603). SBE: self-breast examination

**Variable**	Frequency (n)	Percent (%)
**SBE is a method of screening for breast cancer**		
**Strongly agree**	372	61.7
**Somewhat agree**	143	23.7
**Strongly disagree**	14	2.3
**Somewhat disagree**	6	1.0
**Not indicated**	68	11.3
**SBE should be done only if you feel abnormal around your breast**		
**Strongly agree**	80	13.3
**Somewhat agree**	72	11.9
**Strongly disagree**	402	66.7
**Somewhat disagree**	40	6.6
**Not indicated**	9	1.5
**Postures for SBE are standing in front of mirror, while bathing/lying down**		
**Strongly agree**	356	42.5
**Somewhat agree**	266	44.1
**Strongly disagree**	21	3.5
**Somewhat disagree**	2	0.3
**Afraid of detecting breast cancer so would not practice SBE**		
**Strongly agree**	172	28.5
**Somewhat agree**	48	8.0
**Strongly disagree**	337	55.9
**Somewhat disagree**	42	7.0
**Not indicated**	4	0.7

## Discussion

The present confirm findings from studies conducted in Nigeria over the past years, on BSE and breast cancer among women in the south-east [31], south [29, 32], south-west [33–35] and north [36] of the country. All these studies showed that knowledge on BSE as a screening method for breast cancer and on the right time to carry out BSE was very poor. The only contrast in the present study is that knowledge about postures involved in preforming BSE was good.

Important knowledge deficits can have a detrimental impact on the education of women on screening practices and attitudes of women in the adoption of early detection practices [21]. A correlation may exist between level of education and breast cancer knowledge [37] educational level and marital status as predictors of (CBE) and (BSE) [28,38]. Two studies in Nigeria indicated that education and employment in professional jobs significantly influenced knowledge of breast cancer [21,39]. Our study revealed significant differences between the respondents’ market area, age up to 49 years, marital status and educational level and their knowledge on SBE.

Assessment of the participants’ knowledge, attitude and beliefs showed that majority of the respondents reported that the right time to perform self-breast examination was ‘anytime’ and majority also disagreed that BSE should be done only when they feel abnormal around the breast. Some of the respondents reported that they would not practice BSE because they are afraid of detecting any evidence suggestive of breast cancer. In addition, findings from the study reveal that most of the study population have heard of breast cancer as a disease and self-breast examination as a screening method but there is still inadequate knowledge and understanding of the disease and its screening method. These findings are similar to the study among market women in Abakaliki (south-east Nigeria) by [33], women in south-west Nigeria by [28,33–34], women in Federal Capital Territory of Nigeria by Banning and Ahmed [40].

There is a great need for more awareness campaigns, advocacy to improve the knowledge of self-breast examination directed towards women of low socio-economic status and people at the grassroots levels in the country. This will ensure early detection and intervention to prevent mortality due to breast cancer.

We conclude that knowledge about how and the time to perform BSE among Nigerian women working in the markets in Ibadan, south west Nigeria is very poor, particularly among women who are single, young with a low level of education after controlling for confounders. Similarly, few participants had strong negative beliefs towards breast cancer. Any interventions aimed at improving the knowledge about BSE and breast cancer screening should target these groups. Such interventions should be evaluated to ensure their success in improving women’s health.

The quantitative nature of our study limits the extent to which the information reflects the nuanced views of respondents. A qualitative interview would have allowed a deeper understanding of the perspectives of the respondents.

## Supporting Information

S1 Questionnaire BSE_SEMI_STRUCTURE QUESTIONNAIRE (PDF)
