# Supplementary material for: Knowledge and Beliefs of Breast Self-Examination and Breast Cancer among Market Women in Ibadan, South West, Nigeria
Source: PLoS One. 2015 Nov 25;10(11):e0140904. doi: 10.1371/journal.pone.0140904 (PMC4659560; doi:10.1371/journal.pone.0140904)
Supplement: S1 Questionnaire — (PDF) [file pone.0140904.s001.pdf]

CONSENT FORM

My name is Mrs Oladimeji Kelechi, a postgraduate student of the department of Epidemiology, medical statistics and environmental health, Faculty of Public Health, University of Ibadan. I am carrying out a study on “Knowledge, Attitude and practices of Breast Self Examination (SBE) among women at Ibadan south west community.

I will be using questionnaire to ask some questions relevant to knowledge attitude and practises of Self Breast Examination as early screening tool for breast cancer among women in this community. There may be no direct benefit to you but I do know that the information you provide will help health practionners learn more information on breast self examination in your community.

Taking part is voluntary and you are free to decline consent to take part and also the right to withdraw at any given time you choose. I will appreciate your giving your consent to take part in this study.

Consent Agreement

I have been detailed about the research or have had it translated into language I understand. I have also talked it over with the doctor to my satisfaction. I understand that my participation is voluntary. I know enough about the purpose and benefits of the research study to judge that I want to take part in it. I understand that I may freely stop being part of this study at any time.

DATE: ____________________ SIGNATURE or THUMBPRINT: ______________________

NAME: ____

WITNESS’ SIGNATURE (if participant thumbprints only): _________________________

WITNESS’ NAME (if participant thumbprints only): ________________________________

____

Investigator

BREAST SELF EXAMINATION AMONG MARKET WOMEN

(A) SOCIDEMOGRAPHIC CHARACTERISTICS

1. Serial number: ....
2. Name: ....Phone Number..................................
3. Address: ....
4. How old are you? ....(Age at last birthday)Years
5. Marital Status...
6. Educational Level...
7. What are you selling...
8. How many years you been selling……………………………………………………………......
9. Motherhood (1)Yes (2) No
10. Number of Children……………………………………………………………………………
11. Age at first pregnancy…………………………………………………………………………
12. Age at first menstruation………………………………………………………………………

(B) KNOWLEDGE

1. Do you know how to perform Self breast examination (BSE)?
2. If yes how?
3. How often should BSE be done? a) daily b) weekly c) monthly d) I don't know
4. At what age should BSE be started? ....years
5. When is the right time for a woman to perform BSE? a)before menstruation b)mid cycle c)anytime d)during menstruation

6. Please tick what you think is suitable.

i.) Strongly agree (ii.) Somewhat agree (iii.) Somewhat disagree (iv.) Strongly disagree

	Strongly agree	Somewhat agree	Strongly disagree	Somewhat disagree
	i	ii	iii	iv
1.) BSE is a method of screening for breast cancer				
2.) Frequency of BSE once a month is the best				
3.) You should have BSE only when you feel abnormal around your breast				
4.) Postures of BSE are standing in front of a mirror, while bathing, and lying down				
5.) BSE is very good because it helps to detect early stage of breast cancer				
6.) If you detect a lump, blood or fluid from the breast, inverted nipple, breast size change it is possible you have early stage of breast cancer				
7.) If you have family history of breast cancer you should be conscious to practice BSE				
8.) Do you agree that all women on a regular basis should perform BSE?				
9.) Breast should be feel by the three fingers continuing without lifting finger				
10.) BSE regular practises is important to search the early stage of breast cancer				

(C.) ATTITUDE

Please tick what you think is suitable.

i.) Strongly agree (ii.) Somewhat agree (iii.) Somewhat disagree (iv.) Strongly disagree

	Strongly agree	Somewhat agree	Strongly disagree	Somewhat disagree
	i	ii	iii	iv
1.) You can find breast cancer by yourself				
2.) You are afraid that you will detect breast cancer and so you do not want to do BSE				
3.) Screening for abnormality of BSE is important and useful				
4.) Breast self examination is useless				
5.) BSE for early stage of breast cancer is the duty of doctors, nurses and public health officers				
6.) BSE is complicated, a waste of time and does not give accurate results				
7.) Publicity or campaigns motivate you to screen for breast cancer by yourself on a more regular basis				
8.) Having a breast removed because cancer affect woman's appearance and can motivate women to perform BSE to screen for breast cancer early				
9.) When you have a close relative or neighbour with breast cancer, you are more fearful and want to do BSE				
10.) BSE is disgraceful practice for you				

(C) EXPERIENCES, BEHAVIORAL PRECTICES AND BELIEF

Please tick what you think is suitable.

i.) Continually (ii.) Rarely (iii.) Never

	Continually	Rarely	Never
	i	ii	iii
1.)Have you received knowledge about BSE from radio, television or newspaper			
2.)Have received pamphlet on BSE or/and breast cancer			
3.)Did the family or friend recommend BSE for you			
4.)Are you interested in performing BSE			
5.)Have you received information about using three fingers for BSE			
6.)Have you had BSE over the past 12 months			
7.)Have you used pamphlet to guide yourself			
8.) Have been screened by doctors or nurses over the past year			

What is you belief about breast cancer?..
